# GSK3i combinatorial treatments affect CDK4/6 and compensatory pathways in 3D preclinical models of pancreatic neuroendocrine tumors

**DOI:** 10.1242/bio.062358

**Published:** 2026-03-24

**Authors:** Edlira Luca, Igor Shapiro, Huguette Debaix, Kathrin Zitzmann, Katharina Wang, Christoph J. Auernhammer, Felix Beuschlein, Svenja Nölting, Constanze Hantel

**Affiliations:** ^1^ Department of Endocrinology, Diabetology and Clinical Nutrition, University Hospital Zurich (USZ) and University of Zurich (UZH), 8091 Zürich, Switzerland; ^2^ Department of Medicine IV, LMU University Hospital, LMU Munich, 80336 Munich, Germany; ^3^ Interdisciplinary Center of Neuroendocrine Tumors of the GastroEnteroPancreatic System (GEPNET-KUM, ENETS Center of Excellence), LMU University Hospital, LMU Munich, 81377 Munich, Germany; ^4^ The LOOP Zurich-Medical Research Center, CH-8091 Zurich, Switzerland; ^5^ ENETS Center of Excellence Zurich, University Hospital Zurich, CH-8091 Zurich, Switzerland

**Keywords:** Pancreatic neuroendocrine tumor, BON-1, GSK3, TNFα, Insulin, Spheroids

## Abstract

Pancreatic neuroendocrine tumors (pNETs) are rare tumors, often detected at advanced stages for which current therapies are mostly unsuccessful. Despite significant progress in the understanding of pathogenic molecular pathways, most single targeted treatments only slow disease progression while patients develop resistance due to compensatory mechanisms. To identify novel combination therapies, we assessed the effects of TNFα, insulin and GSK3 inhibition (GSK3i) in spheroids of the metastatic pNET model BON-1 on subcellular localization and activation of cell cycle components and apoptosis. While cyclin D1 and CDK4 and 6 (cyclin-dependent kinase) stainings, related protein levels and apoptosis were not affected or only weakly affected by single treatments, the combinatorial treatments acted synergistically to induce cell death, as assessed by cleaved caspase-3 immunopositivity. Protein levels of CDK4 and CDK6 were furthermore validated in primary pNET cultures. Compensatory mechanisms under the single treatments might include activation of CDK1/2 and the DNA damage response, as assessed by the activation of phospho-Chk2, since this was perturbed under the combinatorial treatments. Taken together, our work identifies combinatorial treatments that substantially reduce the viability of spheroidal BON-1 cultures and patient-derived primary cultures as potential novel therapies for the treatment of pNETs.

## INTRODUCTION

Pancreatic neuroendocrine tumors (pNET) are rare cancers originating predominantly from the endocrine cells of the pancreas through mostly spontaneous but also hereditary genetic mechanisms (Oronsky et al., 2017). The majority of pNETs are non-functional, grow asymptomatically and are diagnosed at an advanced and metastatic stage (Oronsky et al., 2017). The most prevalent mutations in pNET include tumor-suppressor genes responsible for genomic stability, mitogenic PI3K/AKT/mTOR pathway inhibitors and cell cycle suppressors (Maharjan et al., 2021; Scott et al., 2020), implicating the overactivation of survival and proliferation pathways as mechanisms of tumor growth. However, monotherapies targeting individual pathways have only slowed, but not halted, disease progression in clinical trials, suggesting that combination approaches may target compensatory pathway activation and offer greater efficacy (Aristizabal Prada et al., 2018a,b,c; Grande et al., 2020; Maharjan et al., 2021; Mohan et al., 2024; Nolting et al., 2015, 2017). Therefore, investigating the pathways that sustain pNET survival and proliferation in models that more closely mimic *in vivo* tumor growth, such as multidimensional systems, may help identify effective combinatorial treatments.

Unrestrained proliferation and overactivation of cell cycle components are hallmarks of cancer (O'Leary et al., 2016). Cyclin dependent kinases (CDKs) drive cell cycle progression through the activation of specific transcriptional programs, accomplished by CDK4/6 in G_1_, CDK2 in S phase and CDK1 during mitosis (Matthews et al., 2022). Their activity is positively regulated by cyclins, which accumulate at different phases of the cycle, and inhibited by checkpoint proteins, which safeguard DNA/chromatin integrity and trigger cell cycle arrest. Additionally, the cyclin D-CDK4/6 complex couples cell cycle to cellular metabolism and REDOX balance (Cairns et al., 2011; Franco et al., 2020; Wang et al., 2017), whereas cyclin D1 links cell cycle to cellular migration (Fuste et al., 2016). Accordingly, hyperactivity of cyclin D-CDK4/6 is a feature of most cancers (Chung, 2004; O'Leary et al., 2016), including pNETs (Scott et al., 2020; Shi et al., 2017; Tang et al., 2012), while cyclin D1 specifically correlates with metastasis and serves as a diagnostic marker (Drobnjak et al., 2000; Fuste et al., 2016; Guo et al., 2003). Although therapeutic inhibition of CDK4/6 has been successful in some cancers (O'Leary et al., 2016), others are resistant due to genomic rearrangements and possible compensatory behavior of CDK1/2 (Goel et al., 2022; Malumbres et al., 2004; Santamaria et al., 2007; Tetsu and McCormick, 2003). In pNET specifically, the CDK4/6 inhibitor palbociclib was unsuccessful as a monotherapy in clinical trials (Grande et al., 2020). Therefore, pinpointing resistance-associated pathways would greatly contribute to the successful implementation of combinatorial therapeutic strategies.

The activity of CDKs is overseen by checkpoint proteins, which ensure proper integrity, replication and segregation of DNA/chromosomes. Although genomic instability drives cancer, failure to properly address DNA damage acquired over multiple replication cycles or due to therapeutic agents leads to cell death (Matthews et al., 2022). The checkpoint kinases Chk1 and Chk2 are activated by DNA sensors and orchestrate the cellular response by stimulating transcriptional programs and phosphorylating downstream substrates involved in DNA repair, cell cycle arrest and apoptosis (Antoni et al., 2007). During G_1_, the protein kinase ATM (ataxia telangiectasia mutated) activates Chk2 following its recruitment to double-stranded DNA breaks by phospho-H2AX, while protein kinase ATR (ataxia telangiectasia and Rad3 related) recruits and activates Chk1 to replication stress during S phase. The checkpoint kinases share multiple downstream substrates and might even compensate for each other (Antoni et al., 2007; Takai et al., 2002). Most notably, they stabilize and stimulate the function of the transcription factor p53, which in turn exerts transcriptional control over DNA repair, cell cycle components and apoptosis (Aylon and Oren, 2016; Chen, 2016). Additionally, the p53 protein is involved in the regulation of numerous cellular pathways, thereby coordinating the cellular stress response (Aylon and Oren, 2016).

Extracellular stimuli, such as mitogens or cytokines, regulate cellular proliferation by directly affecting the expression and activity of cell cycle components. For example, activation of the insulin/PI3K/AKT pathway positively regulates not only cyclin D and CDK4/6, but Chk1 activity as well (Saltiel, 2021; Selvarajah et al., 2015). Additionally, the prominent inflammatory cytokine TNFα can stimulate transcription of cell cycle proteins, such as cyclin D1 and CDK2 (Baud and Karin, 2009; Mercogliano et al., 2021), in addition to inducing DNA damage and apoptosis (Ababneh et al., 2024; Ben-Baruch, 2022; Yan et al., 2006). A common target and partial mediator of the effects of TNFα and insulin on cancer cell survival is GSK3, a kinase that phosphorylates a vast array of substrates with the purpose of inhibition or ubiquitinylation (Aristizabal Prada et al., 2018b; Balkwill, 2009; Hermida et al., 2017; Maurer et al., 2014; Vlotides et al., 2014). Activation of GSK3 in pNET preclinical models contributes to survival and therapy resistance (Aristizabal Prada et al., 2018b; Mohan et al., 2024), while inhibition of GSK3 induces cytotoxicity (Thapa et al., 2023).

TNFα, insulin and GSK3 have been linked to aggressiveness and metastasis in pNETs (Scott et al., 2020). We have previously shown in BON-1 spheroids, a preclinical metastatic model of pNET (Townsend et al., 1993), that TNFα, insulin (Ins) and inhibition of GSK3 (GSK3i) single treatments do not affect Ki67 index in such a multi-dimensional setting, while combinatorial treatments of GSK3i+TNFα and GSK3i+Ins drastically reduce proliferation (Nolting et al., 2025). The strengths of multidimensional models have been recognized in recent years, since they are more resistant to therapeutic treatments, can acquire invasive traits and display similar characteristics to tumors *in vivo*, at the molecular, genomic and structural levels (Luca et al., 2023; Mehta et al., 2012). Interestingly, inhibition of GSK3 profoundly affected cell–cell interactions and caused the complete dissociation of the spheroids, thereby permitting TNFα and insulin to exert their effects on comparably scattered cells in the combinatorial conditions (Nolting et al., 2025). Here, we investigated the impact of these treatments on the regulation of cell cycle components and compensatory drug resistance mechanisms in BON-1 spheroids and patient-derived GEP-NET primary cultures.

## RESULTS

**Fig. 1.**
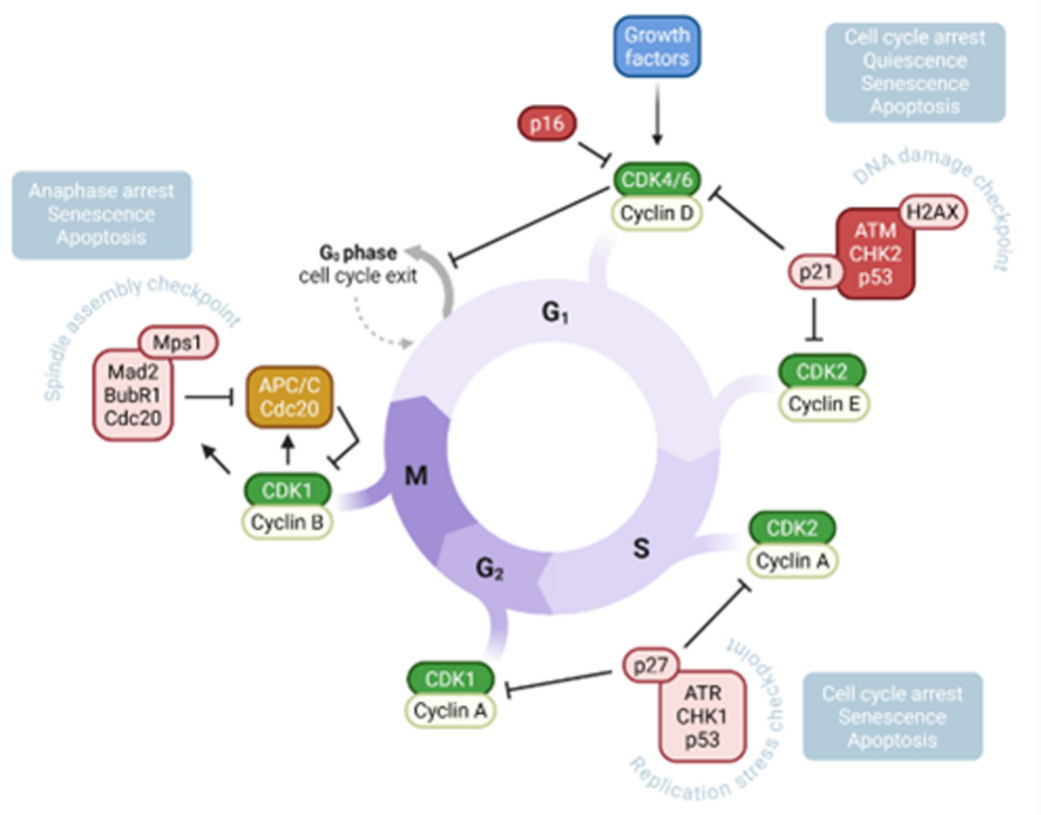
**Regulation of cell cycle.** CDK-cyclin complexes drive progression through the different phases of the cell cycle. The DNA damage checkpoint, replication stress checkpoint and spindle assembly checkpoint inhibit the function of CDK-cyclin complexes to maintain genomic integrity. Created in BioRender by Luca, E. (2026) https://BioRender.com/i84brsc. This figure was sublicensed under CC-BY 4.0 terms.

**Fig. 2.**
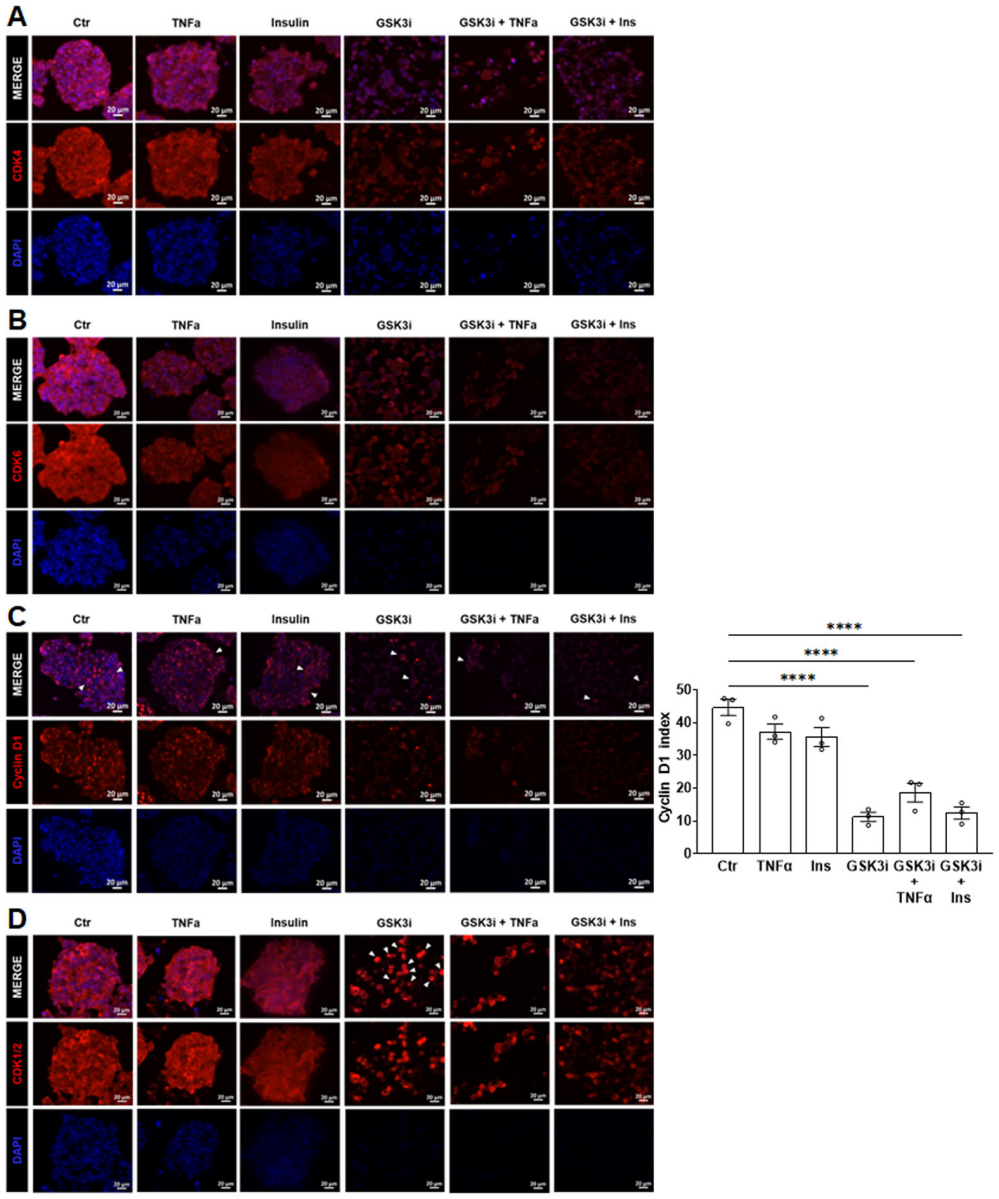
**Immunohistochemistry was performed for CDK4 (A), CDK6 (B), cyclin D1 (C) and CDK1/2 (C).** Nuclear indices are reported for cyclin D1 (*n*=3) and its presence in both the nucleus and cytosol is highlighted by white arrows. Scale bars: 20 µm; statistical analysis performed with ANOVA with Dunnett's multiple comparison to control. *P<0.05, **P<0.01, ****P<<0.0001.

Firstly, we evaluated the subcellular distribution of various regulatory proteins of the cell cycle in BON-1 spheroid cultures (Fig. 1A). CDK4 and CDK6, implicated as the main drivers of tumor proliferation, localized to both the nucleus and cytosol in all conditions (Fig. 2A,B). Immunopositivity was reduced in the spheroids treated with GSK3i and the combinatorial conditions GSK3i+TNFα and GSK3i+Ins as compared to untreated. Consistently, western blot data from monolayer BON-1 cells showed the strongest downregulation of CDK4 in response to GSK3i alone and in combinatorial treatment with Ins, but of CDK6 after combinatorial treatment only (Fig. S2). However, these effects could have not solely explained previously detected additive therapeutic differences observed under combinatory treatments compared to single agents (mainly GSK3i alone). The formation of a complex between either CDK4 or CDK6 with D-type cyclin is essential for their activity and function in the cell cycle. Consistent with the role of cyclin D1 in proliferation and migration, nuclear and cytoplasmatic cyclin D1 immunopositivity were detected (white arrows) in all conditions (Fig. 2C). The cyclin D1 indices (measured as percentage of positive nuclei compared to total nuclei) were similar in untreated spheroids, TNFα and Ins single treatments. The most significant effects were obtained with GSK3i and the combinatorial treatments, where cyclin D1 indices were drastically reduced compared to untreated spheroids. Since loss of CDK4/6-cyclin D complex can be compensated by other kinases, we next investigated the subcellular distribution of CDK1/2. CDK1/2 localized predominately to the cytosol under basal and TNFα treated conditions, as indicated by the double staining with DAPI (Fig. 2D). Ins caused a weaker and more diffuse localization of the kinases throughout the nucleus/cytoplasm. Importantly, CDK1/2 was predominantly localized to the nucleus under GSK3i, while this effect was partially reversed by the combinatorial treatments. Overall, our experiments revealed dynamics in the subcellular localization of the different cell cycle components in response to the single and combinatorial treatments.

**Fig. 3.**
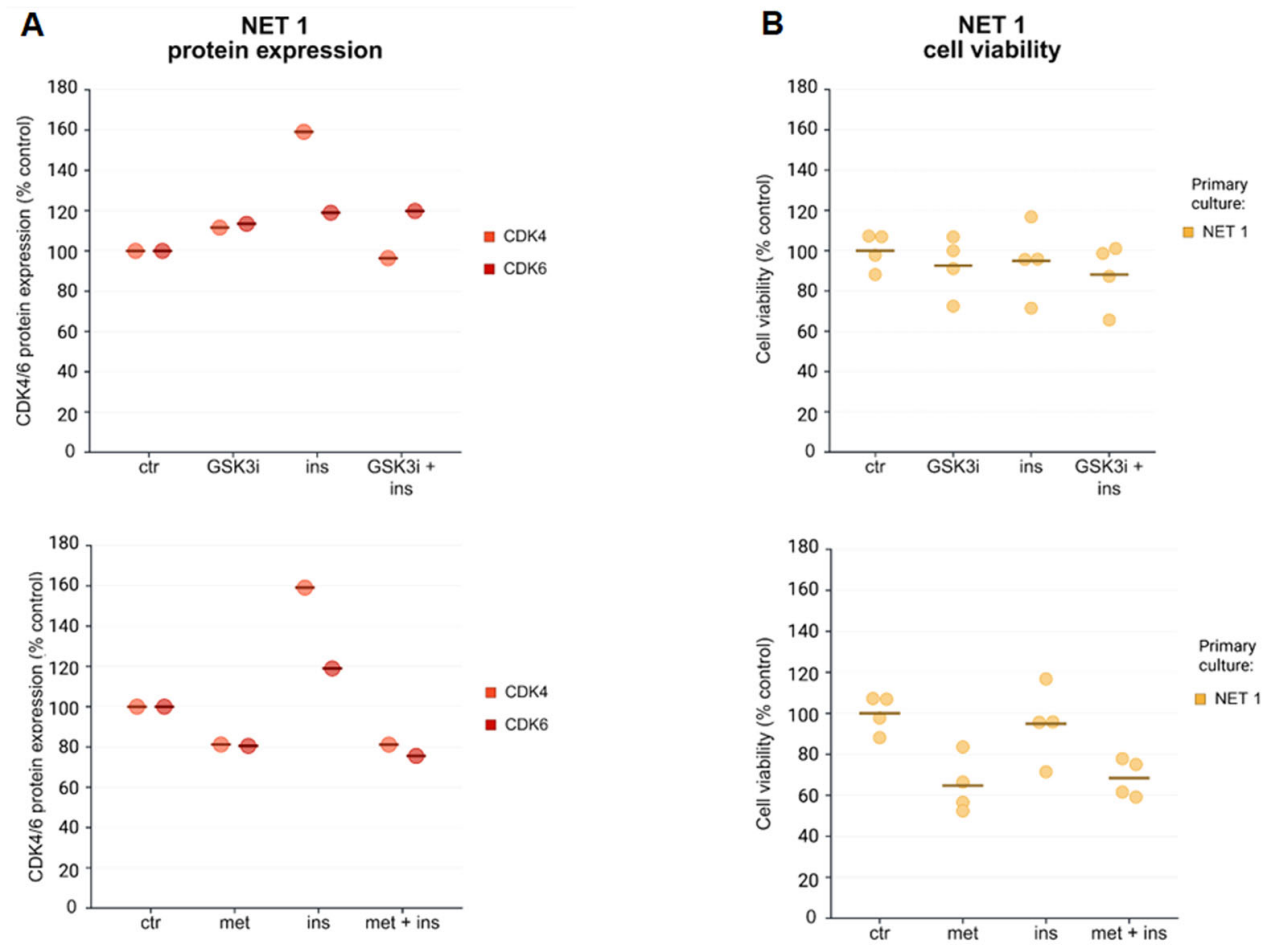
**Representative case study.** (A) Patient-derived pNET primary culture cells (NET1) were incubated with Ins, GSK3i or GSK3i+Ins (upper figure) and Ins, metformin or metformin+Ins (lower figure) before protein expression of CDK4 and CDK6 was measured using JESS Simple Western blotting (*n*=1). (B) Patient-derived pNET primary culture cells (NET1) were incubated with Ins, GSK3i or GSK3i+Ins (upper figure) and Ins, metformin or metformin+Ins (lower figure) for 72 h before cell viability was assessed. Created in BioRender by Wang, K. (2026) https://BioRender.com/sbkv1iu. This figure was sublicensed under CC-BY 4.0 terms.

**Fig. 4.**
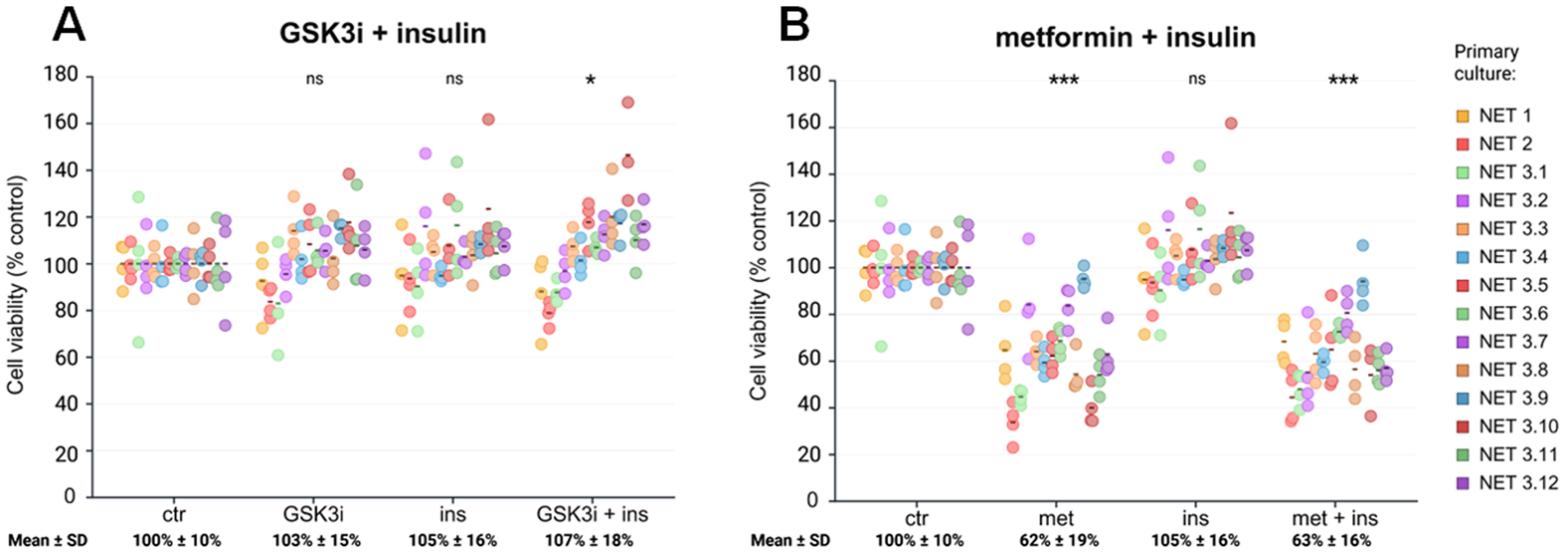
**Statistically powered cohort validation.** Patient-derived NET primary culture viability in response to (A) Ins, GSK3i, GSK3i+Ins or (B) metformin, Ins, metformin+Ins combination treatment (*n*=14: *n*=2 pNETs, *n*=12 different abdominal lymph node metastases of a CUP-NET from the same patient). ns, not significant, *P<0.05, ***P<0.001. Created in BioRender by Wang, K. (2026) https://BioRender.com/sbkv1iu. This figure was sublicensed under CC-BY 4.0 terms.

To support these findings in a complementary translational model, we exemplarily quantified CDK4/6 protein levels in one pNET patient-derived primary culture as a representative case study (NET1; *n*=1), treated with Ins, GSK3i and the combination of both (Fig. 3A, Fig. S1). Surprisingly, and contrary to the BON-1 spheroids and monolayer cells (used as a control), the expression of CDK4/CDK6 in the patient-derived pNET cells after single or combination treatment with Ins and GSK3i remained unchanged or was even increased (Fig. 3A, Fig. S1). Since we have previously shown that the antitumor activity of GSK3i and of the anti-diabetic drug metformin is mediated through common effects on GSK3/Ins signaling in pNET cell lines and patient-derived primary cultures (Nolting et al., 2025; Vlotides et al., 2014), we substituted metformin for GSK3i and investigated CDK4/CDK6 expression in the same experimental setting. Indeed, we could show that metformin alone decreased CDK4/6 expression in the patient-derived pNET primary cells (Fig. 3A, Fig. S1, *n*=1) and in BON-1 monolayers (Figs S2-S4). Stronger CDK4/6 suppression in response to metformin, compared to GSK3i, correlated well with a stronger decrease of patient-derived pNET cell viability (Fig. 3B). Consistently, we have now reconfirmed our findings in a statistically powered cohort validation including two patient-derived pNETs (NET1, NET2) and 12 lymph node metastases of a CUP NET (NET3.1 - NET3.12) (Fig. 4). Metformin led to an overall significant decrease of cell viability (*P*<0.001; Fig. 4B). To provide evidence supporting transferability of these results to 3D BON-1 spheroids, we treated one spheroid each (*n*=1) with 10 mM and 50 mM metformin as proof-of-principle. Consistent with the results in two-dimensional (2D) culture, 10 mM metformin strongly reduced cell viability in BON-1 spheroids (to ∼40% versus control after 9 days of incubation) while 50 mM metformin almost completely abolished cell viability (Fig. S5). The stronger efficacy of metformin, compared to GSK3i alone, was previously published by our group for midgut and pNET patient-derived primary cultures (Nolting et al., 2025). Additionally, since the antitumor effects of metformin have been extensively tested before in a variety of different NET tumor models (Nolting et al., 2025; Vlotides et al., 2014), we decided not to pursue these experiments in more detail.

**Fig. 5.**
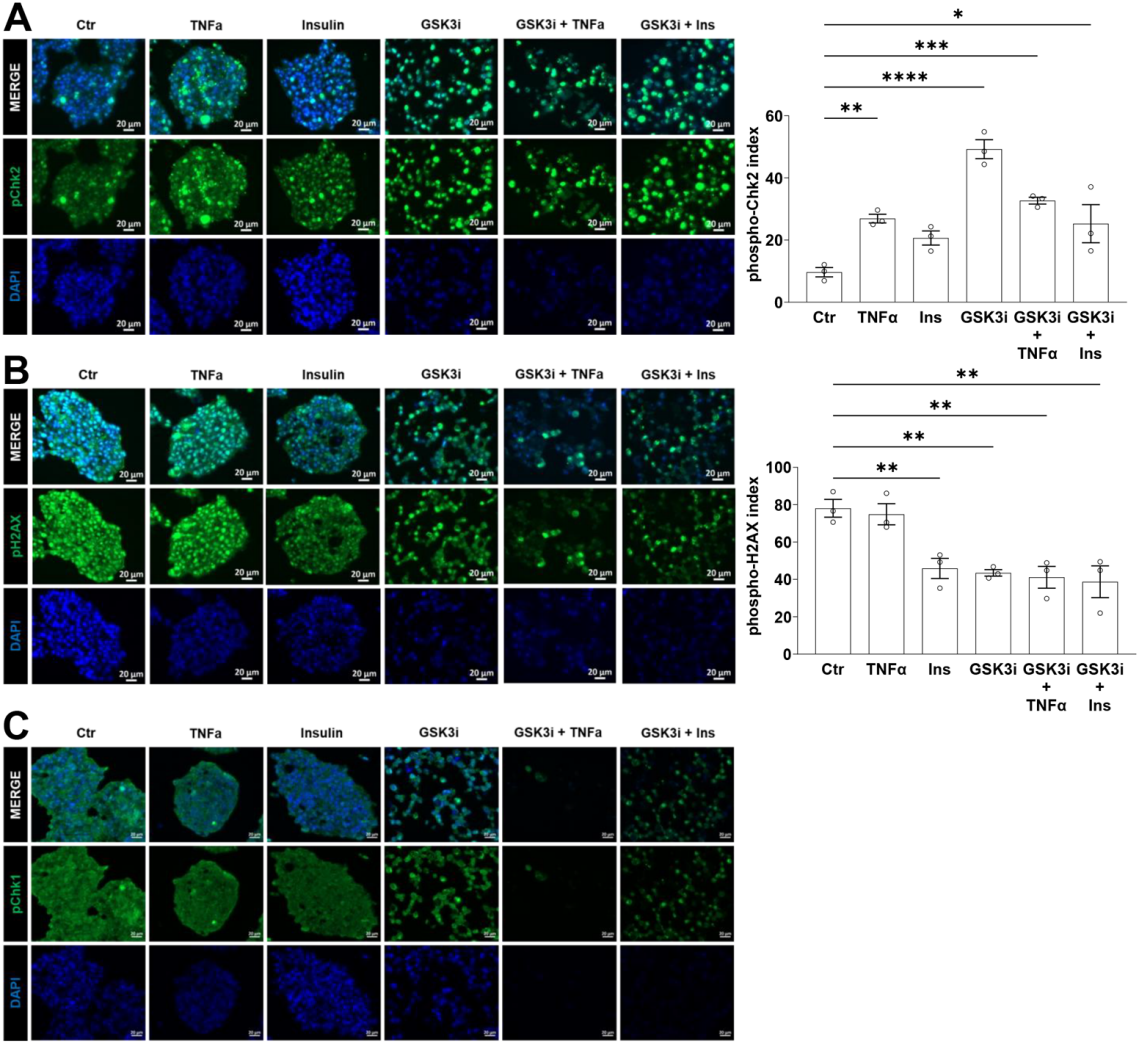
**Immunohistochemistry was performed for phospho-Chk2 (A), phospho-H2AX (B) and phospho-Chk1 (C).** Nuclear indices were calculated for phospho-Chk2 and phspho-H2AX. Scale bars: 20 µm; statistical analysis performed with ANOVA with Dunnett's multiple comparison to control. *P<0.05, **P<0.01, ****P<0.0001.

The activity of cell cycle kinase effectors is supervised by checkpoint proteins which ensure adequate DNA integrity, replication and segregation (Fig. 1A). The DNA damage checkpoint, comprised of ATM and Chk2, is activated by single and double-stranded breaks and induces cell cycle arrest by inhibiting CDK4/6 and CDK2. Baseline activation of Chk2 was present in untreated and Ins-treated spheroids as assessed by the phospho-Chk2 index. Moreover, TNFα and GSK3i significantly increased nuclear phospho-Chk2 compared to untreated spheroids (Fig. 5A). Interestingly, phospho-Chk2 index was reduced in both combinatorial conditions, GSK3i+TNFα and GSK3i+Ins, versus GSK3i alone. Thus, the above described differently regulated cell cycle dynamics were reflected in the activation of Chk2 in the single, but not combinatorial, conditions. Next, we analyzed the histone variant H2AX. Phosphorylation of H2AX marks double-stranded DNA breaks and the subsequent recruitment of DNA repair machinery, including pChk2. In accordance with the baseline activation of Chk2, untreated spheroids exhibited already high phospho-H2AX signal (Fig. 5B). Interestingly, phospho-H2AX did not further increase, but remained elevated under TNFα. In contrast, under all other treatments, phospho-H2AX index was significantly decreased. These results suggested that the effectiveness of damage repair mediated via phospho-H2AX is increased, but maybe not involved solely in the activation of DNA repair. Aside from the DNA damage checkpoint, replication stress during S phase activates ATR and Chk1 proteins, which inhibit the function of CDK1/2 to induce cell cycle arrest (Fig. 1A). Phospho-Chk1 was not robustly activated under any conditions (Fig. 5C). Therefore, BON-1 spheroids might experience more DNA damage than replication stress in response to the treatments.

**Fig. 6.**
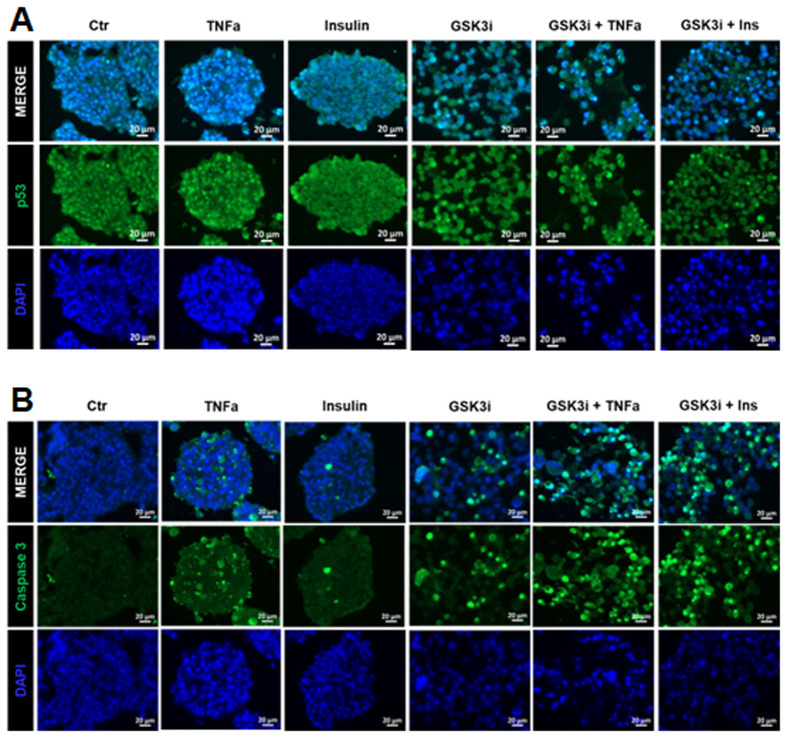
**Immunohistochemistry for p53 (A) and cleaved caspase-3 (B).** Scale bars: 20 µm.

To assess in more detail the changes downstream of these checkpoints, we analyzed the localization of p53 and activation of cleaved caspase-3. Stabilization and nuclear accumulation of p53 downstream of both pCHK2 and pCHK1 transcriptionally regulates DNA repair, cell cycle arrest and apoptosis (Fig. 1A). In untread and TNFα single treatment, p53 was prominently stabilized in the nucleus, as assessed by the double staining of p53 and DAPI, while it was more diffuse throughout cytoplasm/nucleus under Ins treatment (Fig. 6A). Under GSK3i single or combinatorial treatments, p53 was often re-localized to the cytoplasm/nucleus as well, although some cells displayed prominent nuclear signal. Moreover, dotted signals of both DAPI and p53 in the combinatorial conditions indicate strong DNA damage. Thus, we observed a treatment-dependent response in the (de)-stabilization of p53 signal and its nuclear to cellular re-distribution, consistent with decreased CDK4/6, cyclinD1 and peaks in pCHK2 and nuclear intensity and localization of CDK1/2 under GSK3i, indicating potential compensatory mechanisms compared with the combinatorial treatments. Since unresolved DNA damage leads to chromosomal instability and eventually the activation of apoptosis, appropriate effects might accumulate and could be visualized by cleaved caspase-3 levels, which is downstream of both checkpoints (Fig. 6B). In untreated as well as insulin treated spheroids, cleaved caspase-3 was not readily detectable. In contrast, cleaved caspase-3 was most prominent detectable upon both combinatorial treatments.

## DISCUSSION

The overexpression of cyclin D1 and CDK4/6 is thought to be the driving force of uncontrolled cell division in many cancers, including pNETs (Chung, 2004; O'Leary et al., 2016; Scott et al., 2020; Shi et al., 2017; Tang et al., 2012). However, resistance to CDK4/6 inhibitors often arises, due to genomic determinants and possible compensation from CDK1/2 effectors (Goel et al., 2022; Malumbres et al., 2004; O'Leary et al., 2016; Santamaria et al., 2007; Tetsu and McCormick, 2003). Correspondingly, we show that single treatments with TNFα, Ins, GSK3i can effectively decrease expression of CDK4/6 but overall mildly affect apoptosis due to compensatory mechanisms supporting cell cycle progression. BON-1 spheroids were mainly affected by the combinatorial treatments, GSK3i+TNFα and GSK3i+Ins, two conditions where inhibition of CDK4/6 expression and the lack of potential compensatory mechanisms as detected upon single TNFα (nuclear p53) and GSK3i (nuclear CDK1/2) treatment were associated with a drastic activation of cleaved caspase-3.

The importance of CDK4/6 in fueling cell cycle progression in the aggressive BON-1 spheroids is underscored by its subcellular translocation under pro-tumorigenic conditions. Ins favored stronger nuclear immunopositivity of CDK4, and to a lesser extent CDK6, over their cytoplasmic presence, potentially triggering the transition from G1-to-S phase in the spheroids (Matthews et al., 2022). Consequently, the distribution of CDK1/2 became more diffuse and evenly localized between nucleus and cytoplasm under Ins treatment. Although more experiments are necessary to understand the effects of Ins on the expression of CDK proteins, their nuclear presence is consistent with progression of cell cycle and the role of Ins as a mitogen (Bowers et al., 2015). Ins also reduced the activation of the DNA damage response, reflected in reduced phospho-H2AX, and the redistribution of p53 from nucleus to cytoplasm as assessed by the diffuse staining. The role of p53 in cell cycle arrest is linked to its actions as a transcription factor, although p53 is implicated in many cellular processes that are DNA-independent (Aylon and Oren, 2016) and cytoplasmic localization would be associated with these pathways. These results are consistent with the importance of CDK4/6 in driving cell cycle.

We also demonstrate that single anti-tumorigenic treatments that compromise CDK4/6 expression trigger compensatory mechanisms possibly rooted in genomic determinants and CDK1/2 effectors (Goel et al., 2022; O'Leary et al., 2016; Santamaria et al., 2007; Tetsu and McCormick, 2003). BON-1 spheroids treated with TNFα showcase the complex and divergent roles played by this cytokine in cell death and survival (Ababneh et al., 2024; Ben-Baruch, 2022). Under TNFα treatment, localization of cyclin D1 and CDK4/6 was shifted to favor the nucleus over the cytosol, consistent with reports of their regulation by TNFα (Balkwill, 2009; Schmitz and Kracht, 2016). Together with the expression of CDK1/2, TNFα positively affected compensation and cell cycle progression (Malumbres et al., 2004; Tetsu and McCormick, 2003). Consistent with activation of TNFα-induced damage repair (Yan et al., 2006), elevated levels of phospho-Chk2 were associated with pronounced nuclear immunopositivity of its target p53 as compared to untread spheroids. Nuclear p53 exerts transcriptional regulation over DNA repair and cell cycle arrest (Chen, 2016). Consistent with the DNA damage response and cell cycle arrest being protective mechanisms (Matthews et al., 2022), activation of Chk2 was disproportionally larger than activation of cleaved caspase-3, indicating that the majority of cells were protected from apoptosis. Therefore, protective mechanisms might compensate for CDK4/6 under TNFα treatment. Similar results were obtained with the anti-tumorigenic condition GSK3i, which is known to negatively affect cell survival (Aristizabal Prada et al., 2018b). Although inhibition of GSK3 reduced cyclin D1 index, consistent with previous reports (Aristizabal Prada et al., 2018b; Diehl et al., 1998; Ougolkov et al., 2005), GSK3 might be a novel regulator of CDK4/6 since immunopositivity was reduced; however, additional protein measurements would be necessary for proper investigation. Importantly, GSK3i was the only condition where CDK1/2 localized predominantly to the nucleus. Therefore, the reduction in CDK4/6 signal might be compensated by nuclear CDK1/2, in accordance with published reports (Malumbres et al., 2004; Tetsu and McCormick, 2003). We infer that reduced CDK4/6 by anti-tumorigenic single treatments might be readily compensated and partially buffered by the DNA damage response. One limitation of the study is the investigation of only cyclin D1, while investigation of cyclins D2 and D3 remain to be addressed.

However, the combinatorial treatments GSK3i+TNFα and GSK3i+Ins negatively regulated CDK4/6 signal as well as any compensatory mechanisms, since their synergistic effects inflicted widespread apoptosis, as assessed by cleaved caspase-3. Accordingly, localization of CDK1/2 was predominantly cytosolic under both combinatorial treatments, implying failure to activate the cell cycle machinery. Similarly, there is low activation of the DNA damage response, as assessed from decreased phospho-H2AX, decreased phospho-Chk2, and reduced nuclear immunopositivity of p53. We speculate that DNA integrity is severely compromised by the synergistic effects of the combinatorial treatments, and the lack of response from the DNA damage response pathway results in elevated levels of cleaved caspase-3 and apoptosis. Therefore, in contrast to the modest effects of the single treatments, the combinatorial treatments synergistically affected both cell cycle and cell survival. Our results are in line with the notion that checkpoint proteins contribute to therapy resistance (Advani et al., 2015; Pilie et al., 2019) and that elevated genomic instability increases the mutational burden and leads to apoptosis (Antoni et al., 2007).

In contrast to our findings in BON-1 spheroids and monolayer, CDK4/6 expression was not decreased in the exemplarily investigated pNET patient-derived primary culture (NET1) in response to GSK3i or combination treatment. These contrary effects of GSK3i or GSK3i/insulin in the BON-1 model versus the patient-derived pNET primary culture might be explained by a much higher proliferation index (Ki67 around 70%) of the BON-1 cell spheroids versus a Ki67 of only 2% (grading G1) of the investigated patient-derived pNET primary culture. The high Ki67 index renders BON-1 cells much more susceptible to cell cycle disruption, compared to the G1 pNET. This is consistent with no relevant effects of GSK3i or GSK3i/Ins combination treatment on patient-derived G1/G2 pNET cell viability and indicates therapy resistance. In contrast, the anti-diabetic drug metformin led to a CDK4/6 reduction in the exemplarily investigated NET1. Consistently, compared to GSK3i or Ins/GSK3i, metformin led to a stronger and highly significant cell viability decrease in the patient-derived NET primary cultures (*n*=2 pNETs, *n*=12 different abdominal lymph node metastases of a CUP-NET from the same patient), which has, in part, also been published previously (Nolting et al., 2025). On the one hand, our findings confirm that GSK3i and metformin act through common signaling pathways, as previously shown by our group (Nolting et al., 2025; Vlotides et al., 2014), and both affect the cell cycle. On the other hand, the data emphasize that the testing of drugs in different pNET models including highly proliferative BON-1 spheroids and low-grade (G1/G2) patient-derived primary cultures is complementary.

As discussed so far, our experiments indicate modulation of the immunopositivity of p53 in BON-1 spheroids under the different conditions. In untreated as well as TNFα and GSK3i single treatments, p53 staining appears to be more pronounced in the nucleus as compared to the Ins single treatment and the combinatorial conditions where the staining is more diffused. Although more experiments are necessary, we speculate that nuclear p53 together with phospho-Chk2 could contribute under these conditions to DNA repair and cell survival, while therapeutic combinations might lack such a potential rescue mechanism thereby leading to the observed increased apoptosis under these conditions (Chen, 2016; Harper et al., 1993; Aylon and Oren, 2016). Moreover, our results question the notion that p53 is non-functional in BON-1, due to the presence of a point mutation (Vandamme et al., 2015) and deficiencies in cell cycle arrest of monolayer BON-1 cultures (Capodanno et al., 2021). Certain mutations do not completely abrogate the function of p53 but rather affect its ability to regulate specific pathways (Brady et al., 2011; Li et al., 2012). Additionally, although p53 is mutated in the majority of cancers, mutant p53 can also act in pro-tumorigenic capacity (Kastenhuber and Lowe, 2017). Therefore, additional studies of p53 are necessary to clarify its true nature in BON-1 spheroids.

## MATERIALS AND METHODS

### Culturing, fixation and paraffin embedding of BON-1 tumor spheroids

BON-1 spheroids were formed by plating 25,000 cells per well in Sphericalplates 5D (Kugelmeiers, Erlenbach, Switzerland) and culturing them over 6 days. Treatment with TNFα (0.05 µg/ml, GIBCO, Thermo Fisher Scientific), Ins (3 nM, Chemie Brunschwig, Basel, Switzerland), AR-A014418 (20 µM, Lucerna-Chem, Lucern, Switzerland) and their combinations started on day 7 and lasted for 72 h, with three biological replicates per condition. General plating procedure, fixation and paraffin embedding was done as previously described (Bornstein et al., 2022).

### Culturing of BON-1 tumor spheroids for three-dimensional (3D) cell viability assay

BON-1 spheroids were formed by plating 1000 cells per well in 96-well Black Round Bottom Ultra-Low Attachment Surface Spheroid Microplates (Corning, Glendale, USA) and culturing them over 10 days. Treatment with metformin (10 mM and 50 mM) started on day 11 and lasted for 6 days and 9 days, respectively. The metformin-containing medium was renewed every 72 h. Cell viability was measured using CellTiter-Glo 3D assay (PROMEGA, Madison, USA) according to the manufacturer's instructions.

### Immunofluorescence and immunohistochemistry

Analysis of immunofluorescence images was conducted from images of technical replicates from one well per condition. Sections were deparaffinized, subjected to antigen retrieval in sodium citrate buffer, blocked and incubated overnight at 4°C with primary antibody. Primary antibodies are listed in Table 1. The next day, secondary antibody conjugated to Alexa-fluorophores (Table 1) was applied for 1 h at room temperature. In the case of multiple stainings, primary antibodies were applied sequentially. DAPI was used at final concentration 1 µg/ml. Images were acquired with a Zeiss Axio Imager M2 equipped with the AxioCam 512 color, using ZEN 3.3 software (Carl Zeiss, Oberkochen, Germany). Three images per condition (*n*=3) were analyzed with ImageJ and nuclear index was calculated as percentage of positive nuclei compared to total nuclei.

**Table 1.** Primary and secondary antibodies used for immunofluorescence in the study

Antibody	Ordering information	Dilution
Cyclin D1	55506, Cell Signaling Technology (Danvers, MA, USA)	1:500
CDK4	12790, Cell Signaling Technology (Danvers, MA, USA)	1:500
CDK6	sc-7961, Santa Cruz Biotechnology (USA)	1:100
CDK 1/2	sc-53219, Santa Cruz Biotechnology (USA)	1:100
phospho-Chk2	2197, Cell Signaling Technology (Danvers, MA, USA)	1:200
phospho-Chk1	2348, Cell Signaling Technology (Danvers, MA, USA)	1:50
phospho-H2AX	2577S, Cell Signaling Technology (Danvers, MA, USA)	1:1000
p53	M7001, Agilent DAKO (USA)	1:50
Cleaved caspase-3	9661, Cell Signaling Technology (Danvers, MA, USA)	1:300
Donkey anti-goat Alexa Fluor 568	A11057, Invitrogen (Waltham, MA, USA)	1:500
Goat anti-mouse Alexa Fluor 488	A21121, Invitrogen (Waltham, MA, USA)	1:500
Goat anti-mouse Alexa Fluor 568	A11004, Invitrogen (Waltham, MA, USA)	1:500
Goat anti-rabbit Alexa Fluor 488	A11034, Invitrogen (Waltham, MA, USA)	1:500

### Culturing, treatment and viability of 2D BON-1 cell culture and GEP-NET primary cultures

14 patient-derived gastroenteropancreatic (GEP) NET primary cultures were established from three patients (*n*=14, *n*=2 pNETs, *n*=12 different abdominal lymph node metastases of a CUP-NET from the same patient, Table 2). Following written informed consent, fresh tumor tissues were obtained from surgical specimens at the University Hospitals Zurich and Munich as part of NeoExNET approved by the respective local ethics committees (project numbers BASEC 2017-00950, 152-10). Briefly, primary cultures were generated and incubated with Ins (500 pM), AR-A014418 (10 µM), metformin (10 mM), or a combination of Ins (500 pM)+AR (10 µM) or Ins (500 pM)+metformin (10 mM) for 72 h. Metabolic activity was measured with Cell Titer Blue cell viability assay (Promega, Madison, USA) using a GLOMAX plate reader (Promega) according to the manufacturer's instructions. The experiments were performed in quadruplicates. The data was summarized as the percentage of control of the mean (±s.d.).

**Table 2.** Patient and tumor characteristics of the patient-derived primary cultures

Patient ID Labor	Sex	Age (yrs)	Grading (WHO)	Tumor type	Localization	Tumor size	Metastatic	Mutation	MIB1/Ki-67
NET 1	f	70	NET G1	Pancreatic NET	Primary tumor	2.3 cm	No	n/a	2%
NET 2	f	48	NET G1	Pancreatic NET	Primary tumor	8 mm	No	n/a	n/a
NET 3.1	f	30	NET G2	NET of unknown origin	Metastasis (lymph node) mesenteric	5× lymph node together 10,5×6×7, max. 6.5 cm	Yes	Negative	3%
NET 3.2					Metastasis (lymph node) iliaca interna	2× lymph node together 6.5×4.5×3, max. 5.1 cm	Yes	Negative	3-5%
NET 3.3					Metastasis (lymph node) distal vena cava	2× lymph node together, max. 6.3 cm	Yes	Negative	n/a
NET 3.4					Metastasis (lymph node) interaorto caval	lymph node-conglomerate 14×8×6	Yes	Negative	n/a
NET 3.5					Metastasis (lymph node) truncus coeliacus	11× lymph node together 11×9×4, max. 6 cm	Yes	Negative	n/a
NET 3.6					Metastasis (lymph node) arteria mesenterica superior left	5.5×3.5×4.5	Yes	Negative	n/a
NET 3.7					Metastasis (lymph node) interaortocaval posterior	3× lymph node together 6×4×3.5, max. 5 cm	Yes	Negative	n/a
NET 3.8					Metastasis (lymph node) splenic hilum	5.8×3.5×2.5	Yes	Negative	n/a
NET 3.9					Metastasis (lymph node) pancreas head and hepatic hilum	11.5×7.5×6	Yes	Negative	n/a
NET 3.10					Metastasis (lymph node) preduodenal	2.5×1.6×1.5	Yes	Negative	n/a
NET 3.11					Metastasis (lymph node) retroduodenal processus uncinatus	15×8×6.8	Yes	Negative	n/a
NET 3.12					Metastasis (lymph node) ligamentum treitz	13.2×8.1×7.0	Yes	Negative	n/a

### JESS Simple Western™

Fully automated western blotting (JESS Simple Western™; ProteinSimple, San Jose, CA, USA) was used to measure relative protein expression levels. Whole cell protein lysates were prepared and examined according to the manufacturer's instructions. The antibodies (Abs) used were: CDK4 (#12790, 1:50) and CDK6 (#30483, 1:30), both from Cell Signaling Technology. The secondary Ab (anti-rabbit HRP) and enhanced chemiluminescence (ECL) reagents were used according to the kit's instructions (anti-rabbit detection module chemiluminescence; ProteinSimple^®^, Bio-Techne). The Ab diluent, washing buffer, plates and capillary cartridges used were derived from the 12-230 kDa separation module (ProteinSimple^®^, Bio-Techne). For normalization of specific protein expression, RePlex was used before total protein expression was detected (RePlex™ reagent kit and Protein Detection Module for Chemiluminescence based total protein assays; both ProteinSimple®, Bio-Techne).

### Image analysis and quantification

JESS Simple Western™ data were analyzed using Compass for Simple Western software (6.3.0). Images from the high dynamic range 4.0 were used for the analysis, and peaks were automatically detected. Both peak height and area were analyzed.

### Statistical analysis

Immunofluorescent images were analyzed with ImageJ. For comparisons between treatment groups, one-way ANOVA with Dunnett's multiple comparison was performed in GraphPad Prism 10. Statistical analysis of the GEP-NET primary cultures was conducted with one-way ANOVA and *post hoc* Dunnett's test using IBM SPSS Statistics, Version 31.0.0.0 (IBM Corp. Armonk, NY, USA, released 2025).

## Supplementary Material

10.1242/biolopen.062358_sup1 Supplementary information
