# Supplementary material for: GSK3i combinatorial treatments affect CDK4/6 and compensatory pathways in 3D preclinical models of pancreatic neuroendocrine tumors
Source: Biol Open. 2026 Mar 24;15(3):bio062358. doi: 10.1242/bio.062358 (PMC13054929; doi:10.1242/bio.062358)
Supplement: Supplementary information [file biolopen-15-062358-s1.pdf]

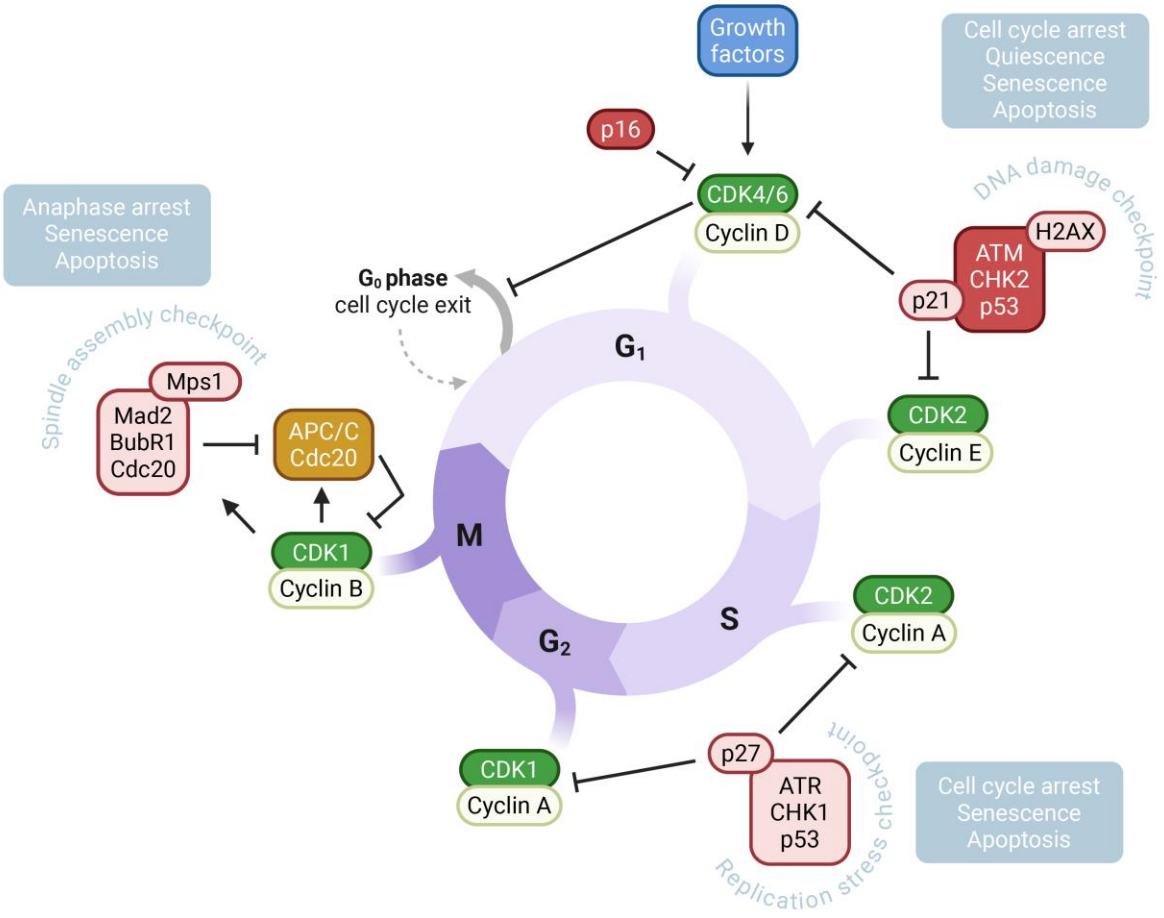

Fig. S1. Lane view of CDK4/6 JESS analysis in patient-derived pNET primary culture cells (NET1) after incubation with GSK3i, metformin, insulin, GSK3i + insulin or metformin + insulin.

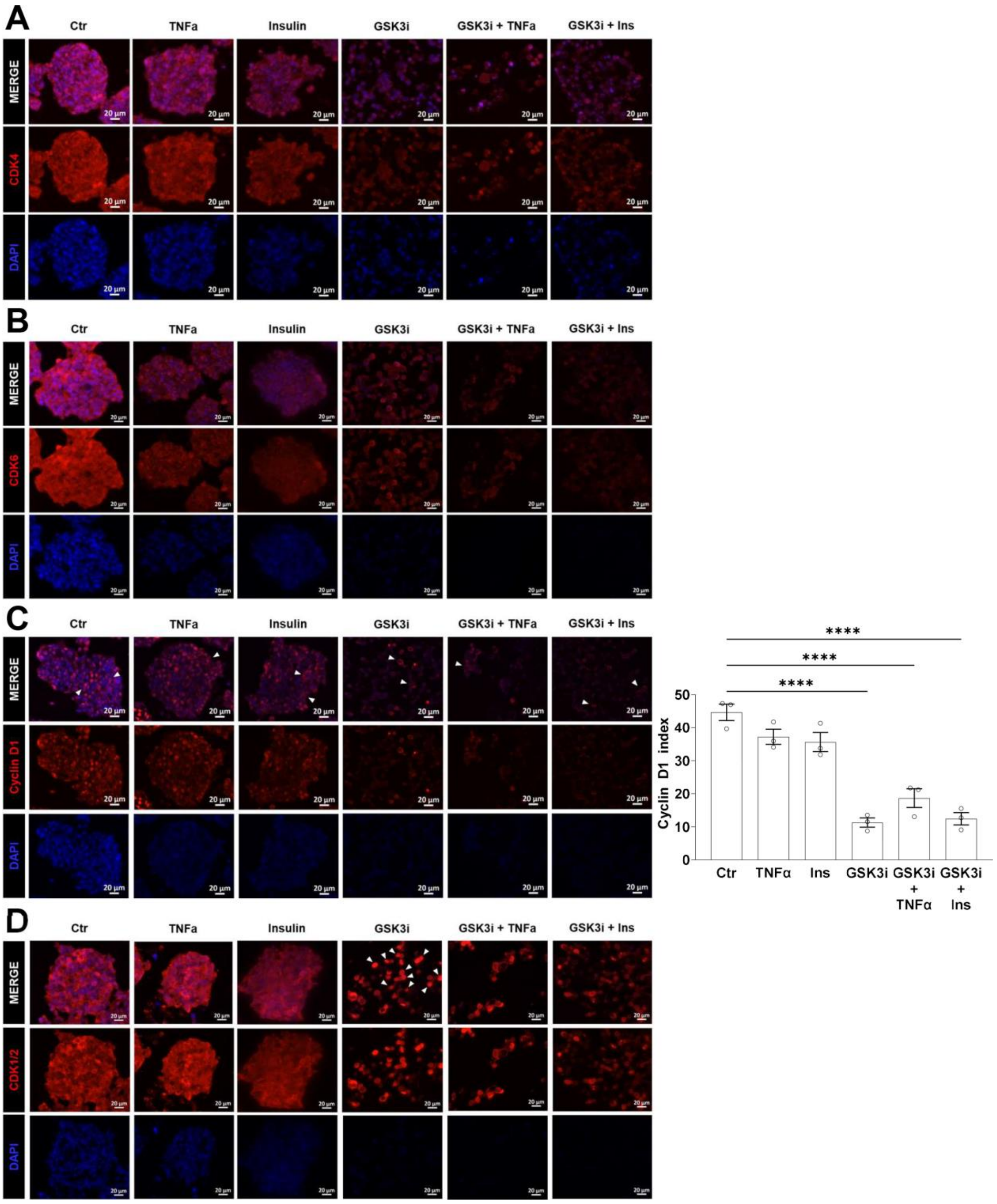

Fig. S2. Protein expression of CDK4/6 in BON-1 monolayer was measured after incubation with insulin, GSK3i or GSK3i + insulin (A) and insulin, metformin or metformin + insulin using JESS Simple Western blotting (n=2) (B). Figure created with BioRender.

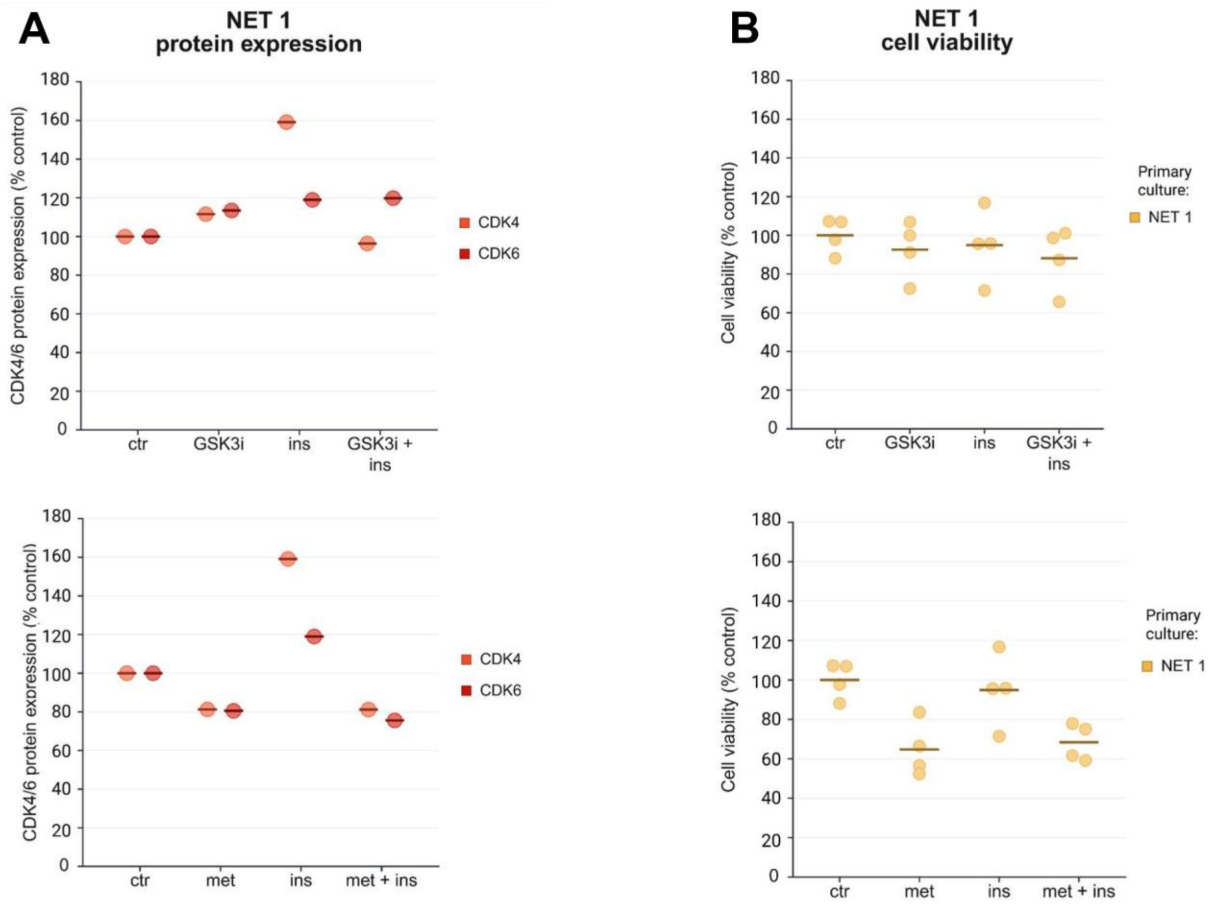

Fig. S3. Lane view of CDK4/6 JESS analysis in BON-1 cells after incubation with GSK3i, metformin, insulin, GSK3i + insulin or metformin + insulin.

Biology Open • Supplementary information

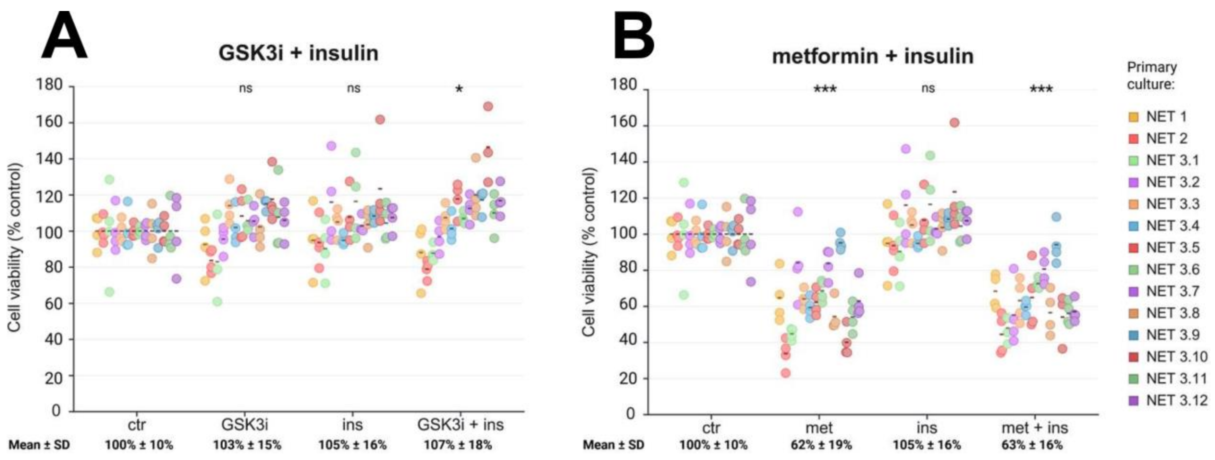

Fig. S4. Lane view of CDK4/6 JESS analysis in BON-1 cells after incubation with GSK3i, metformin, insulin, GSK3i + insulin or metformin + insulin (repetition of the experiment).

Biology Open • Supplementary information

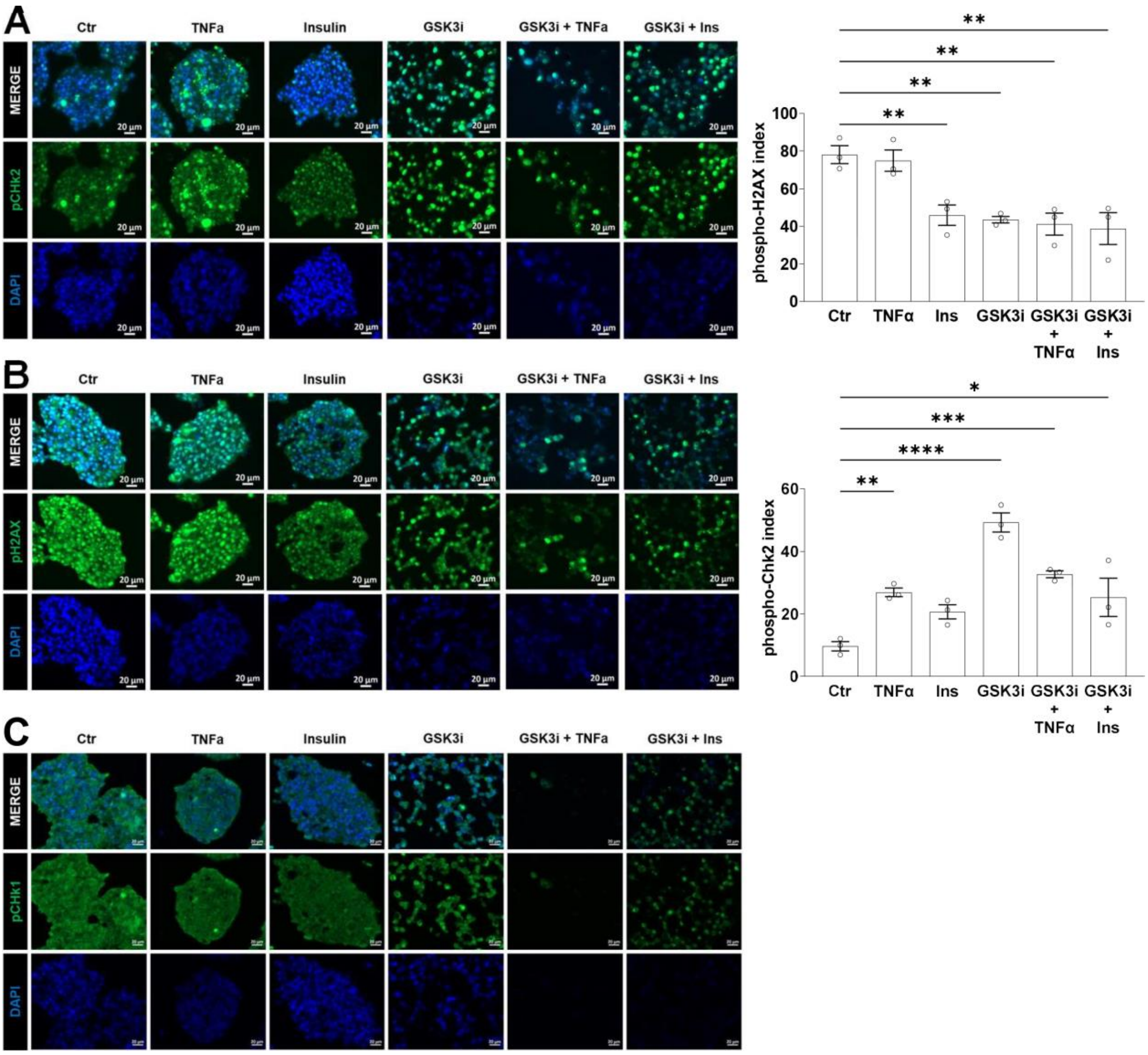

Fig. S5. Cell viability of BON-1 spheroids after incubation with 10mM or 50 mM metformin for 6 days and 9 days, respectively (n=1): Figure created with BioRender.

Table S1. Primary and secondary antibodies used for immunofluorescence in the study.

Antibody	Ordering information	Dilution
Cyclin D1	55506, Cell Signaling (Danvers, MA, USA)	1:500
CDK4	12790, Cell Signaling (Danvers, MA, USA)	1:500
CDK6	sc-7961, Santa Cruz Biotech (USA)	1:100
CDK 1/2	sc-53219, Santa Cruz Biotech (USA)	1:100
phospho-Chk2	2197, Cell Signaling (Danvers, MA, USA)	1:200
phospho-Chk1	2348, Cell Signaling (Danvers, MA, USA)	1:50
phospho-H2AX	2577S, Cell Signaling (Danvers, MA, USA)	1:1000
p53	M7001, Agilent DAKO (USA)	1:50
Cleaved Caspase-3	9661, Cell Signaling (Danvers, MA, USA)	1:300
Donkey anti-goat Alexa Fuor 568	A11057, Invitrogen (Waltham, MA, USA)	1:500
Goat anti-mouse Alexa Fuor 488	A21121, Invitrogen (Waltham, MA, USA)	1:500
Goat anti-mouse Alexa Fuor 568	A11004, Invitrogen (Waltham, MA, USA)	1:500
Goat anti-rabbit Alexa Fuor 488	A11034, Invitrogen (Waltham, MA, USA)	1:500

Biology Open • Supplementary information

Table S2. Patient and tumor characteristics of the patient-derived primary cultures.

Patient ID Labor	*Sex*	*Age (yrs.)*	*Grading (WHO)*	*Tumor type*	*Localisation*	*Tumorsize*	*Metastatic*	*Mutation*	*MIB1 / Ki-67*
NET 1	f	70	NET G1	Pancreatic NET	Primary tumor	2.3 cm	No	n/a	2%
NET 2	f	48	NET G1	Pancreatic NET	Primary tumor	8 mm	No	n/a	n/a
NET 3.1	f	30	NET G2	NET of unknown origin	Metastasis (lymph node) mesenteric	5x lymph node together 10,5x6x7, max. 6.5 cm	Yes	Negative	3%
NET 3.2					Metastasis (lymph node) iliaca interna	2x lymph node together 6.5x4.5x3, max. 5.1 cm	Yes	Negative	3-5%
NET 3.3					Metastasis (lymph node) distal vena cava	2x lymph node together, max. 6.3 cm	Yes	Negative	n/a
NET 3.4					Metastasis (lymph node) interaorto caval	lymph node-conglomerate 14x8x6	Yes	Negative	n/a
NET 3.5					Metastasis (lymph node) truncus coeliacus	11x lymph node together 11x9x4, max. 6 cm	Yes	Negative	n/a
NET 3.6					Metastasis (lymph node) arteria mesenterica superior left	5.5x3.5x4.5	Yes	Negative	n/a
NET 3.7					Metastasis (lymph node) interaortocaval posterior	3x lymph node together 6x4x3.5, max. 5 cm	Yes	Negative	n/a
NET 3.8					Metastasis (lymph node) splenic hilum	5.8x3.5x2.5	Yes	Negative	n/a
NET 3.9					Metastasis (lymph node) pancreas head + hepatic hilum	11.5x7.5x6	Yes	Negative	n/a
NET 3.10					Metastasis (lymph node) preduodenal	2.5x1.6x1.5	Yes	Negative	n/a
NET 3.11					Metastasis (lymph node) retroduodenal processus uncinatus	15x8x6.8	Yes	Negative	n/a
NET 3.12					Metastasis (lymph node) ligamentum treitz	13.2x8.1x7.0	Yes	Negative	n/a
